# Response: Commentary: Cognitive Behavioral Therapy vs. Eye Movement Desensitization and Reprocessing for Treating Panic Disorder: A Randomized Controlled Trial

**DOI:** 10.3389/fpsyg.2018.02045

**Published:** 2018-10-24

**Authors:** Ferdinand Horst, Brenda Den Oudsten, Wobbe Zijlstra, Ad de Jongh, Jill Lobbestael, Jolanda De Vries

**Affiliations:** ^1^Department of Psychiatry, St. Elisabeth Hospital, Tilburg, Netherlands; ^2^Department of Medical and Clinical Psychology, Centre of Research on Psychology in Somatic Diseases, Tilburg, Netherlands; ^3^Department of Education and Research, St. Elisabeth Hospital, Tilburg, Netherlands; ^4^Department of Behavioral Science, Academic Centre for Dentistry, University of Amsterdam and VU University, Amsterdam, Netherlands; ^5^School of Health Sciences, Salford University, Manchester, United Kingdom; ^6^Institute of Health and Society, University of Worcester, Worcester, United Kingdom; ^7^Department of Clinical Psychological Science, Maastricht University, Maastricht, Netherlands; ^8^Department of Medical Psychology, St. Elisabeth Hospital, Tilburg, Netherlands

**Keywords:** EMDR (eye movement desensitization and reprocessing), CBT (cognitive-behavioral therapy), RCT (randomized controlled trial), panic disorder (PD), psychotherapy

Perna et al. (2018) wrote a commentary in which they respond to two aspects of our study (Horst et al., 2017). First, they try to downplay the results of our study by suggesting that we did not use a measure of severity. This is surely an example of reading our article the wrong way. In fact, we used a set of valid measures closely related to the severity of panic disorder (PD). Two of these, the Agoraphobic Cognitions Questionnaire (ACQ) and the Body Sensations Questionnaire (BSQ) are among the most popular and well-researched instruments for assessing panic disorder and agoraphobia worldwide. For example, interpreting bodily sensations (as indexed by the BSQ) is commonly considered to play a key role in the dynamics underlying panic disorder in that individuals suffering from panic disorder display a tendency to interpret bodily sensations as an imminent catastrophe, thereby initiating a vicious circle that reinforces panic (e.g., Clark, 1986; McNally, 1994). This is further supported by numerous studies showing that BSQ total scores and scores on measures directly assessing the severity of panic attacks, such as the Panic Attack Questionnaire-Revised (PAQ-R), are significantly associated. For instance, McGinn et al. (2015) reported a correlation of −0.44 between ACQ and PAQ-R and a correlation of 0.40 between the BSQ and the PAQ-R. In addition, a panic-related interpretation bias, as indexed with the ACQ, is not merely predictive of panic attacks, but even of new onsets of panic disorder (Woud et al., 2014). Moreover, the cognitions related to the panic attacks as assessed with the ACQ directly reflect two main DSM-IV criteria of PD, i.e., persistent concerns about having additional attacks and worry about the implications of the attack or its consequences (American Psychiatric Association, 2013). Concerning the BSQ, this questionnaire literally asks patients to indicate how often they experienced the physical symptoms mentioned in the DSM-IV-TR (Frances, 2004).

Furthermore, Perna et al. (2018) argue that our abstract conclusion that EMDR therapy is as effective as CBT for PD patients is overstated. This argument is largely taken out of context. Specifically, this sentence in our abstract was immediately preceded by an overview of the specific outcome measures of this study. These outcome concepts were again specified in the main conclusion of our discussion (i.e., regarding to severity of a wide range of PD symptoms, including anxiety related cognitions, fear of bodily sensations, as well as quality of life).

The second issue raised by Perna et al. (2018), concerns a lack of description of the method used to determine the non-inferiority (NI) margins of outcome measures. As referenced by Perna et al. (2018), NI margins should be based on statistical reasoning as well as clinical judgment. Starting with the clinical judgement, there were no existing comparable studies that could provide information. Therefore, the principle investigator consulted eight licensed clinical psychologists, familiar with the questionnaires and the population of patients with PD, asked how large should the score of a particular questionnaire increase or decrease to indicate that the patient very likely improved or worsened. In addition, concerning statistical reasoning, effect sizes were calculated based on T1 for the entire group. These effect sizes are shown in Table 1.

**Table 1:** Effect sizes for both treatment groups EMDR and CBT together for baseline (T1).

**Outcome**	**ES = delta/*SD***	**ES**
**SYMPTOMS**		
ACQ	5/10.95	0.46
BSQ1	5/12.45	0.40
BSQ2	5/11.05	0.45
MI-ac	8/18.85	0.42
MI-al	8/24.50	0.33
**QOL**		
OQOL	1/3.60	0.28
Physical health	1/2.80	0.36
Psychological health	1/2.51	0.40
Social relationships	1/2.90	0.34
Environment	1/2.40	0.42

*ACQ, Agoraphobic Cognitive Questionnaire; BSQ1, Body Symptoms Questionnaire (amount of fear); BSQ2, Body Symptoms Questionnaire (how often sensations are experienced); CBT, Cognitive Behavioral Therapy; EMDR, Eye Movement Desensitization and Reprocessing; ES, Effect size; MI-ac, Mobility Inventory (when accompanied); MI-al, Mobility Inventory (when alone); QOL, Quality Of Life; OQOL/GH, Overall Quality Of Life and General Health*.

Assuming an effect size of 0.05 *SD* on a QOL score is considered relevant (Norman et al., 2003), all used NI margins are lower. The smaller the NI margin, the more difficult is it to demonstrate non-inferiority. So, according to the 0.5^*^SD-rule, the chosen NI margins are all on the conservative side with regard to non-inferiority testing.

In conclusion, Perna et al. (2018) tried to undermine our results and drew conclusions from our study that were unwarranted. We have conducted our study with the utmost scrutiny.

## Author contributions

FH and JD drafted the manuscript. BO, AdJ, JL, and WZ revised the manuscript for important intellectual content. FH, BO, WZ, AdJ, JL, and JD approved the final version of the manuscript.

### Conflict of interest statement

AdJ reported receiving income for published books or book chapters on EMDR and for training professionals in this method. The remaining authors declare that the research was conducted in the absence of any commercial or financial relationships that could be construed as a potential conflict of interest.
